# Circulating gut microbiota-related metabolites influence endothelium plaque lesion formation in ApoE knockout rats

**DOI:** 10.1371/journal.pone.0264934

**Published:** 2022-05-06

**Authors:** Hsiao-Li Chuang, Chien-Chao Chiu, Ching Lo, Cheng-Chih Hsu, Ju-Yun Liu, Shao-Wen Hung, Shih-Chieh Tsai, Hsiang-Hsuan Sung, Chi-Kuang Leo Wang, Yen-Te Huang

**Affiliations:** 1 National Laboratory Animal Center, National Applied Research Laboratories, Taipei, Taiwan; 2 Division of Animal Industry, Animal Technology Laboratories, Agricultural Technology Research Institute, Miaoli, Taiwan; 3 Department of Chemistry, National Taiwan University, Taipei, Taiwan; Max Delbruck Centrum fur Molekulare Medizin Berlin Buch, GERMANY

## Abstract

Atherosclerosis is the main cause of cardiac and peripheral vessel infarction in developed countries. Recent studies have established that gut microbiota and their metabolites play important roles in the development and progression of cardiovascular disease; however, the underlying mechanisms remain unclear. The present study aimed to investigate endothelium plaque lesion formation in ApoE-deficient rats fed a normal chow diet under germ-free (GF) and specific-pathogen-free (SPF) conditions at various time points. There was no difference in serum cholesterol and triglyceride levels between SPF-rats and GF-rats. Histological studies revealed that the GF-rats developed endothelium plaques in the aorta from 26 to 52 weeks, but this was not observed in SPF-rats. GF-rat coronary arteries had moderate-to-severe endothelium lesions during this time period, but SPF-rat coronary arteries had only mild lesion formation. Immunohistochemical staining showed higher accumulation of CD68-positive and arginase-negative foamy-like macrophages on the arterial walls of GF-rats, and expression of TNF-α and IL-6 in foam cells was only observed in GF-rats. In addition, microbial metabolites, including equol derivatives, enterolactone derivatives, indole-3-propionate, indole-3-acrylic acid, cholic acid, hippuric acid, and isoquinolone, were significantly higher in the SPF group than in the GF group. In conclusion, our results indicate that gut microbiota may attenuate atherosclerosis development.

## Introduction

Atherosclerosis is a chronic disease that can remain asymptomatic for decades, and is the main underlying cause of cardiovascular disease (CVD) [1, 2]. It is associated with dyslipidemia, increased expression of inflammatory factors, and endothelial dysfunction [3]. Apolipoprotein E (ApoE) is a glycoprotein synthesized primarily in the liver that plays a pivotal role in the clearance of chylomicrons and very low-density lipoproteins [4]. ApoE^-/-^ knockout mice exhibit increased low-density lipoproteins and very low-density lipoproteins plasma levels and develop extensive atherosclerotic lesions distributed throughout the aorta [5].

The microbiota has been reported to be linked to the development of atherosclerosis and other metabolic disorders [6, 7]. Gregory et al. reported that atherosclerosis susceptibility can be transmitted by gut microbiota transplantation in mice [7]. When fed a low cholesterol diet, there was an absence of atherosclerotic plaques in specific pathogen free (SPF) ApoE^-/-^ deficient mice compared to that in mice reared under germ free (GF) conditions, suggesting that commensal microflora protected the ApoE^-/-^ mice against the development of lesions [8]. Interestingly, the impact of the gut microbiota on atherosclerosis severity is dietary dependent and is associated with plasma cholesterol levels [6]. When fed a Western diet, *Lachnospiraceae* was significantly enriched in ApoE^-/-^ mice compared to that in wild type mice, which was positively correlated with relative atherosclerotic lesion size in ApoE^-/-^ mice [9].

Microbial metabolites have an indirect effect on atherosclerosis by regulating lipid metabolism and inflammation [10]. For example, a strong association was noted between atherosclerotic plaque and plasma trimethylamine N-oxide (TMAO) levels [7]. Cason et al. found that the concentrations of plasma metabolites, including indole and indole-3-propionic acid, were negatively associated with advanced atherosclerosis [11]. Another study discovered phospholipid-associated molecules, including choline and betaine, associated with atherosclerosis [12]. Human and animal studies suggest that several families of bacteria, including *Deferribacteraceae*, *Anaeroplasmataceae*, *Prevotellaceae* [13], and *Enterobacteriaceae* [14], are involved in trimethylamine (TMA)/TMAO production. According to these studies, microbiota metabolites appear to affect the development of atherosclerosis.

The role of gut microbiota and microbial metabolites in the process of atherosclerosis remains unclear. For example, Wright et al. observed no effect of gnotobiosis on the progression of atherosclerosis in GF ApoE^-/-^ mice compared with that in control mice [15]. Despite Apo E mice have expanded our understanding in atherosclerosis, the translation from mouse to human has not been so persuasive in the field of cardiovascular research including atherosclerosis. The possible reason is in contrast to human atherosclerosis which develops very slowly compared with Apo E mice [16]. CRISPR/Cas9-mediated knockout of *Apoe* in rats. The previously known progression of atherosclerosis in *Apoe* knockout rat is relatively slower than that of mouse, it could be speculated that Apoe knockout rats could be more suitable preclinical animal model to reproduce the normal or pathological background of early stage atherosclerosis in humans [17]. The ApoE^-/-^ rat model can compensate for this defect. The purpose of this study was to use ApoE-deficient rats reared under GF and SPF conditions to determine the effects of gut microbiota and microbial metabolites on the formation of endothelium plaque lesions.

## Materials and methods

### Generation of ApoE knockout rats

**Fig 1:**
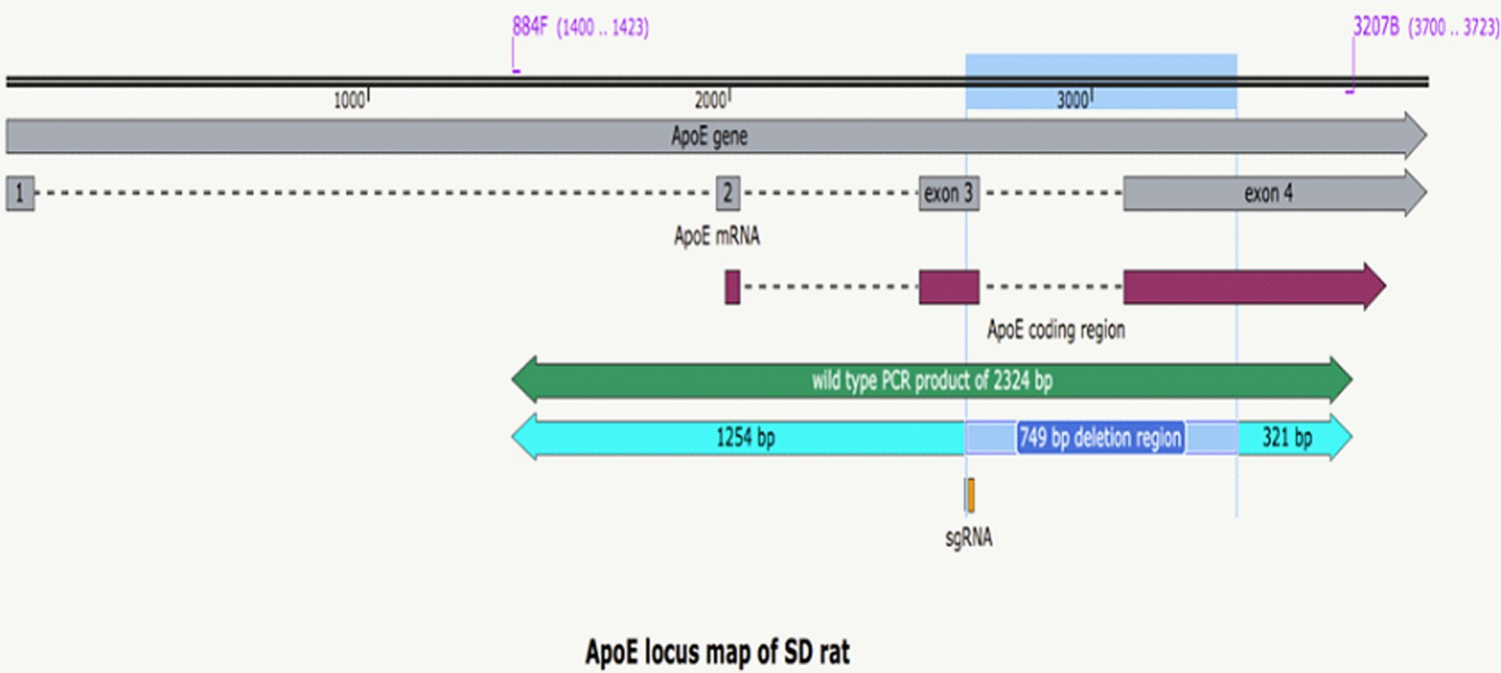
Detailed genetic map of the *ApoE* locus in this study. The *ApoE* gene of SD rats consists of one noncoding exon at the 5′ terminal followed by three coding exons. One single guide RNA (sgRNA, position as indicated) induced deletion from exon 3 to exon 4 was introduced by pronuclear injection of CRISPR/Cas9 components in SD rat embryos. Founder No. 27 carries a 749 bp deletion and was designated as *ApoE*^*em1Narl*^ (mutation indicated at the corresponding region). This results in a truncation of the ApoE protein from amino acid 59 to 175, and was chosen as the functional “null” mutant of the *ApoE* locus. Primers (884F and 3207B) are marked at the corresponding sites that led to a 2324 bp PCR product (1254 bp + 749 bp + 321 bp) in wild type rat, whereas the *ApoE*^*em1Narl*^ mutation resulted in a 1575 bp product (1254 bp+ 321 bp) that is present in the genotype of every pup.

A single guide RNA (sgRNA) with the specific targeting sequence GGGAGCUCUGCAGCUCUUCC and 5′ capped Cas9 mRNA with a 3′-poly-A tail were synthesized in vitro using a MEGAshortscript™ T7 Transcription Kit and mMESSAGE mMACHINE™ T7 Ultra kit, respectively (Ambion, Huntingdon, UK). Pronuclear injection of both sgRNA and Cas9 mRNA was performed on Sprague-Dawley (SD) rat embryos at the single-cell stage to introduce double-strand breaks and potential indel mutations after non-homologous end joining at the corresponding site on chromosome 1. Surviving embryos were implanted surgically to the oviducts of pseudo-pregnant female recipients who gave birth to individual pups. The genomic DNA of each offspring was amplified using polymerase chain reaction (PCR) with specific primers (884F, 5′- ATCTCCAGGAGTATGGGACTGTCG-3′, and 3207B 5′-ATGTCTTCCACTAGCGGCTCGAAC-3′) to screen the integrity of the ApoE loci. While the intact allele signal was found to be 2324 bp, founder No. 27, which carried a specific 753 bp PCR product, was selected for further breeding. The sequencing of a particular genotype (27.1) was characterized as a deletion of 749 bp across exons 3 and 4, resulting in the truncation of ApoE protein from amino acids 59 to 175 (Fig 1). The derived mutant line, strain designation: SD-*ApoE^em1Narl^*/Narl (RMRC No. 23006), was initially bred with wild-type SD rats that maintained *the ApoE^em1Narl^* (null) genotype as a heterozygous state for at least five generations before intercrossing to generate homozygous rats. This backcross breeding process substantially diluted potential off-target effects of CRISPR/Cas9, if any, during the genetic manipulation process at the very beginning of the founder embryo. The resulting heterozygous rats (>N5) were then intercrossed to obtain the experimental group of homozygous and control groups of wild-type littermates under both SPF and GF conditions at the National Laboratory Animal Center (NLAC). To control the sterility of the isolator, the cecum of the foster mother rat from the isolator was evaluated and cultivated after lactation in the presence of aerobic and anaerobic bacteria, mold, and yeast. All animals were maintained on a 12 h light/12 h dark cycle at room temperature (21 ± 2°C), with 55%–65% relative humidity. GF and SPF rats were fed an autoclaved commercial diet (5010 LabDiet, Purina Mills, St. Louis, MO, USA) and sterile water ad libitum. All animal experiments conformed to the guidelines of the Institutional Animal Care and Use Committee (IACUC) of the National Laboratory Animal Center, Taiwan. This study was approved by the IACUC ethics committee under protocols IACUC2014M11 and IACUC2016M03.

### Experimental design

SPF and GF homozygous ApoE^-/-^ rats (n = 3–4, respectively) were exposed to differing microbial conditions to observe the formation of atherosclerosis lesions at three different time intervals of 13, 26, and 52 weeks. The animals were euthanized by CO_2_ asphyxiation followed by exsanguination at 13, 26, and 52 weeks. Blood was drawn by cardiac puncture for clinical chemistry analysis. Tissue samples, including the heart and thoracic aorta, were fixed overnight with 10% neutral buffered formalin for paraffin section preparation and histopathological observation.

### Clinical chemistry

Serum samples were centrifuged at 2700 × *g* for 10 min. Serum total cholesterol (T-CHO) and triglyceride (TG) levels were determined using an automatic analyzer (HITACHI 7080, Hitachi, Tokyo, Japan).

### Histological examination and Elastica–Masson Trichrome (EMT) staining

Sagittal sections of the heart and thoracic aorta were obtained and stained with hematoxylin and eosin (H&E) for histological examination. EMT staining was performed to assess atherosclerotic changes in the aorta. The luminal surface and atherosclerotic plaque areas were compared among the aortic portions in stained cross-sections. The cross-sectional area of plaque in the coronary artery of the heart was measured by NIH ImageJ software version 1.53a (The National Institutes of Health NIH, Bethesda, Maryland, USA) and reported in μm^2^.

### Immunohistochemistry

Paraffin-embedded colon sections were deparaffinized, rehydrated, subjected to antigen retrieval, and incubated with 3% H_2_O_2_ to eliminate endogenous peroxidase activity. Sections were incubated with 10% skim milk to reduce non-specific reactions and incubated overnight at 4°C with anti-rat CD68 (1:100; AbD Serotec, Kidlington, UK), anti-human α-smooth muscle actin (1:100; Dako Cytomation, Glostrup, Denmark), anti-human Von Willebrand Factor (1:100; Dako Cytomation, Glostrup, Denmark), anti-TNF-α (1:200; Novus Biologicals, Colorado, USA), anti-IL6 (1:100; Novus Biologicals, Colorado, USA), and anti-arginase 1 (1:200; Abcam, Massachusetts, USA). The samples were then incubated with horseradish peroxidase-conjugated polymer (HRP Polymer Conjugate, Invitrogen, CA, USA). Signals were detected by adding the chromogenic substrate AEC. Sections were rinsed with deionized water, counterstained with hematoxylin, and mounted for histological analysis.

### Metabolomic profiling

Metabolomic profiling was performed using a protocol described previously by Dunn et al. [18]. Briefly, metabolites were extracted by adding methanol, followed by protein removal by centrifugation. The supernatant was freeze-dried, re-suspended in water, and centrifuged again to avoid precipitation. Each sample (10 μL) of serum was injected into a Dionex Ultimate 3000 ultra-high-performance liquid chromatography (UHPLC) system coupled with a QE plus mass spectrometer (Thermo Fisher Scientific). A 2.1 × 100 mm Acquity BEH 1.7 μm C18 column (Waters) was kept at 40°C. The binary mobile phase consisted of deionized water containing 0.1% formic acid as solvent A and LC-MS grade acetonitrile with 0.1% formic acid as solvent B. The flow rate was 0.25 mL/min with linear gradient elution of over 15 min. For the first minute, solvent B percentage was held at 0%, linearly increased to 100% for the next 7 min, kept constant at 100% for 3 min, and finally returned to 0% in 1 min. Mass spectrometry (MS) data were collected in positive mode with a default top10 data-dependent acquisition method. The mass scan range was set from 70 to 1000 m/z. The activation energy was set at a stepped normalized collision energy (NCE) of 15 and 25. Raw data were processed by Global Natural Products Social Molecular Networking (GNPS) and Compound Discoverer 2.1 (CD 2.1) [19]. Peak detection, alignment, noise filtering, peak area normalization, and statistical analysis were conducted using CD 2.1, whereas compound identification was conducted using both GNPS and CD 2.1. The results from CD 2.1 and GNPS were exported and combined with homemade programs. The principle component analysis score plot was visualized using OriginPro 9.0 (OriginLab Corporation, MA, USA) and the heat maps were visualized using the made4 package [20].

### Statistical analysis

Statistical analyses were performed using GraphPad Prism 6 (GraphPad Software, La Jolla, CA, USA). Values are expressed as the mean ± standard deviation (SD). All statistical comparisons were analyzed using a non-parametric Mann-Whitney U test or one-way analysis of variance with Bonferroni’s correction for multiple comparisons. Statistical significance was set at P < 0.05.

## Results

### Evaluation of systemic effects in ApoE^-/-^ rats under different microbial conditions

Serum biochemistry were measured at each time point (13, 26, and 52 weeks). The TG and T-CHO of GF and SPF wild type (WT) rats and ApoE^-/-^ rats were not significantly different at any age in the GF and SPF groups (Table 1, S1 Table).

**Table 1:** Serum biochemistry of ApoE rats at different ages in GF and SPF rats.

weeks	groups	T-CHO (mg/dL)	TG
			(mg/dL)
**13**	**GF**	973.7±21.0	2392.1±610.5
	**SPF**	907.0±130.1	2192.0±709.2
**26**	**GF**	877.0±6.7	2651.1±335.9
	**SPF**	1064.9±126.2	2422.4±514.9
**52**	**GF**	718.5±94.7	2606.3±587.7
	**SPF**	863.5±132.6	2445.7±328.3

Values are expressed as mean ± SD. T-CHO: total cholesterol; TG: triglycerides.

### Histologic changes in thoracic aorta lesions

**Fig 2:**
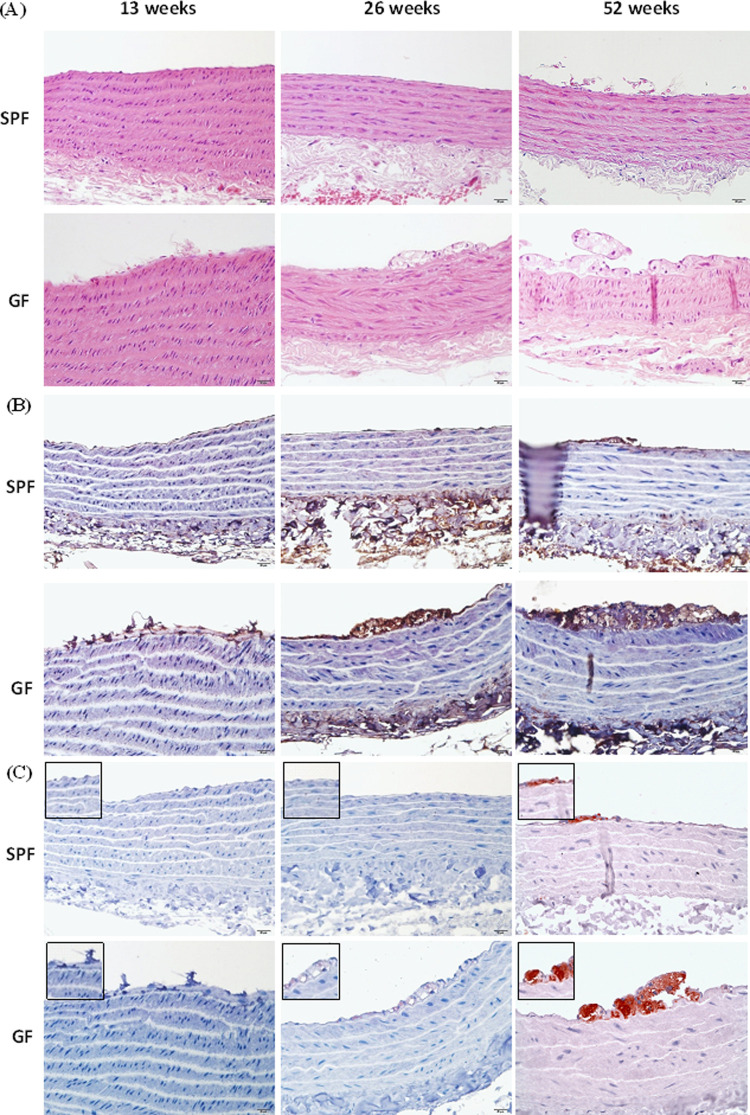
Histopathology and immunohistochemical staining of thoracic aorta lesions. (A) H&E stain. (B) Endothelium injury (Von Willebrand Factor) staining. (C) Macrophage (anti-CD68) staining. GF: germ free, SPF: specific pathogen free.

**Fig 3:**
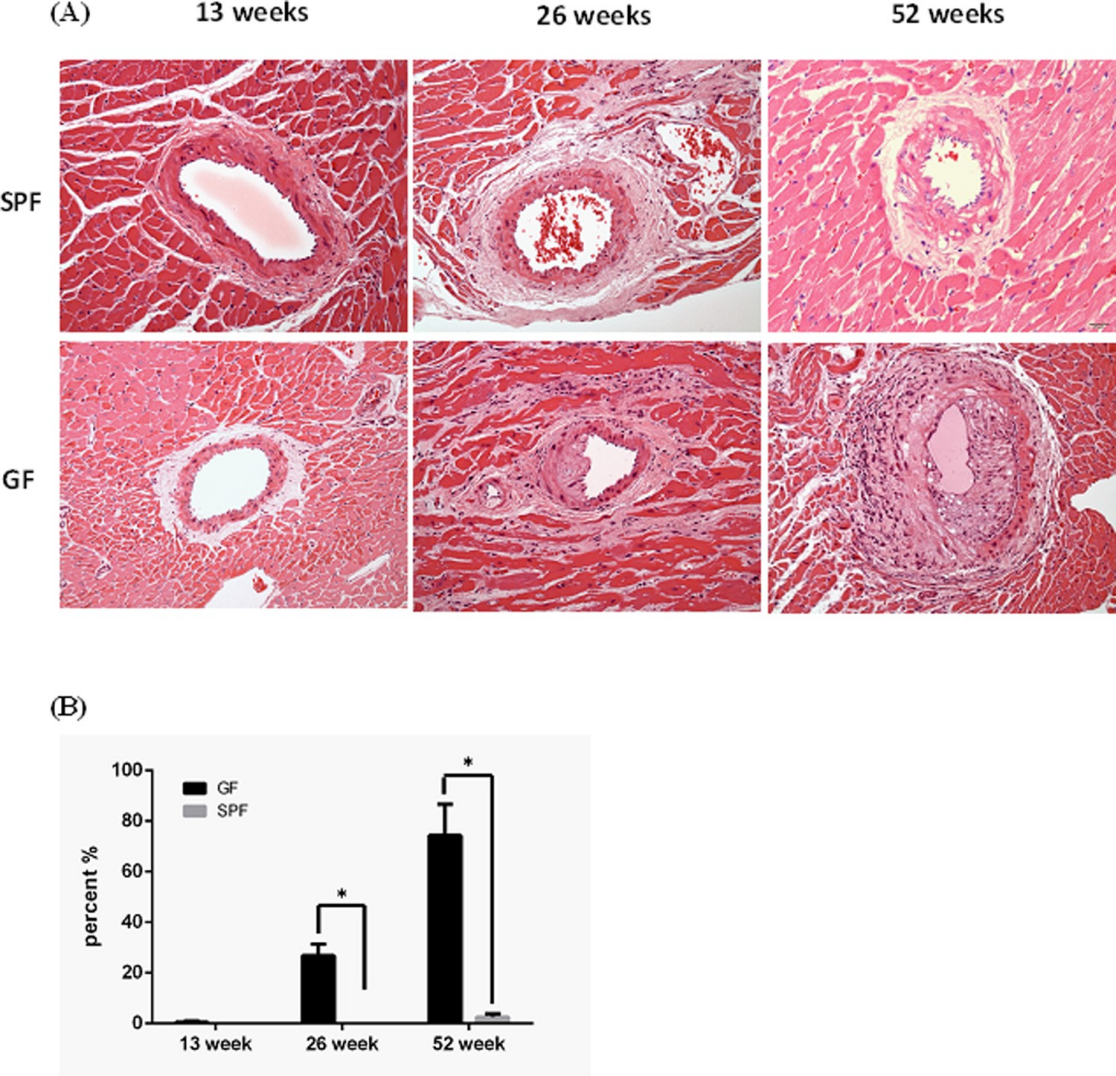
Atherosclerosis lesions of cardiac coronary artery of ApoE^-/-^ rats reared under different microbial conditions. (A) The progress of cardiac atherosclerosis lesions from 12 to 52 weeks. (B) The semi-quantitative analysis of atherosclerotic lesions from 13 to 52 weeks. GF: germ free, SPF: specific pathogen free. Analyzed using Image-J software.

GF-ApoE^-/-^ rats displayed fibrous and cotton-like substances sedimentary on the aortic surface of the vascular endothelium at 13 weeks. The aortic atherosclerosis lesions became fatty streaks at 26 weeks and progressed to fibrous plaques accompanied by a small number of inflammatory cells (mainly foamy cells) at 52 weeks (Fig 2), whilst in SPF-ApoE^-/-^ rats, the early atherosclerotic lesions were barely detected at 13 and 26 weeks. Only mild atherosclerotic lesions (fatty streaks) were observed in the 52-week-old rats. In contrast, moderate to severe atherosclerotic lesions were observed in the cardiac coronary arteries of GF-ApoE^-/-^ rats compared with those in the cardiac coronary arteries of SPF-ApoE^-/-^ rats from 26 to 52 weeks of age (Fig 3A). A semi-quantitative analysis of atherosclerotic lesions from to 13–52 weeks is shown in Fig 3B.

### Detection of cell composition in aortic endothelium plaque lesions by immunohistochemistry

**Fig 4:**
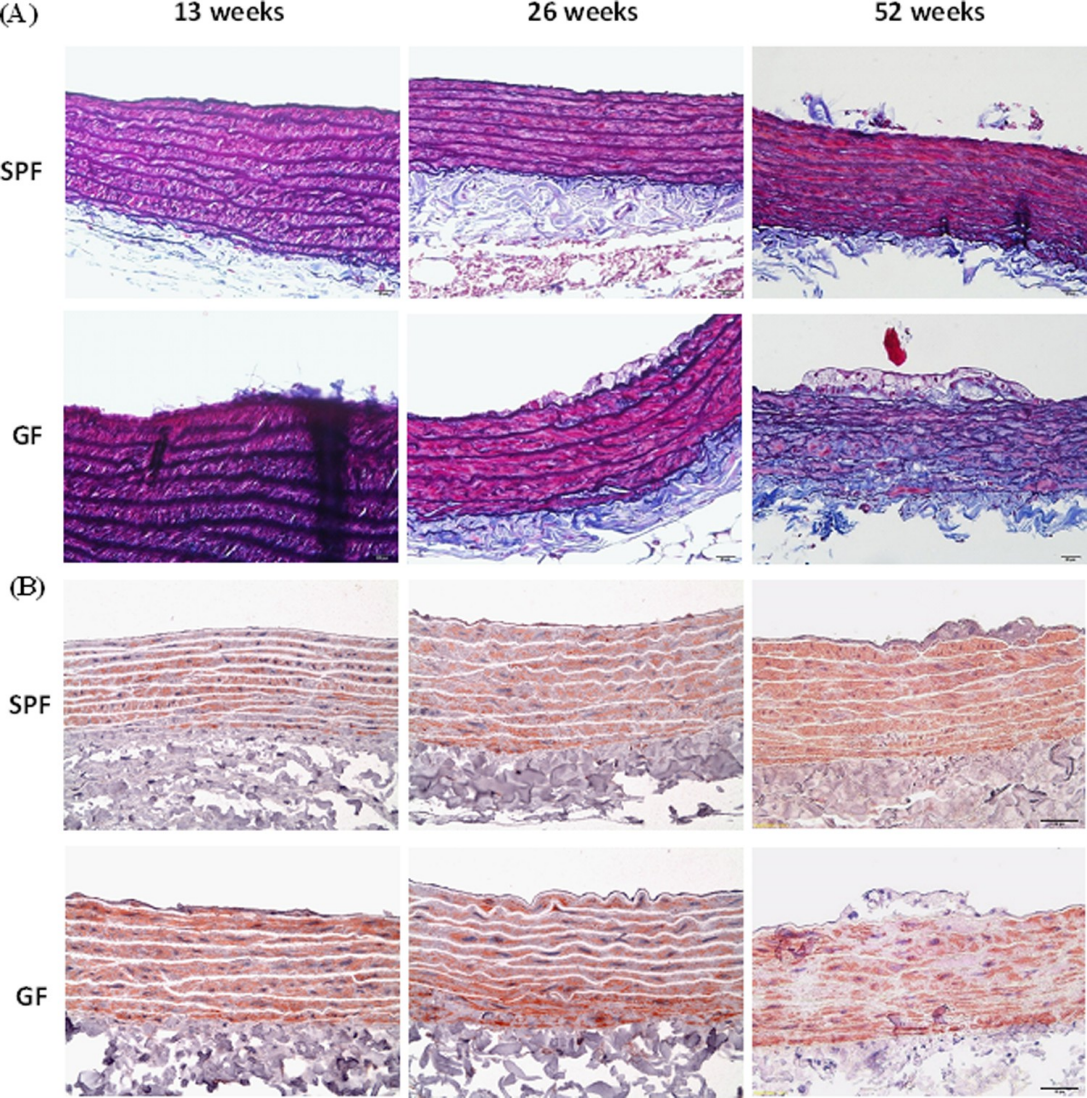
Histopathology and immunohistochemical staining of thoracic aorta lesions. (A) Elastic fiber (Elastica–Masson’s trichrome) stain, and (B) α-smooth muscle actin stain. GF: germ free, SPF: specific pathogen free.

**Fig 5:**
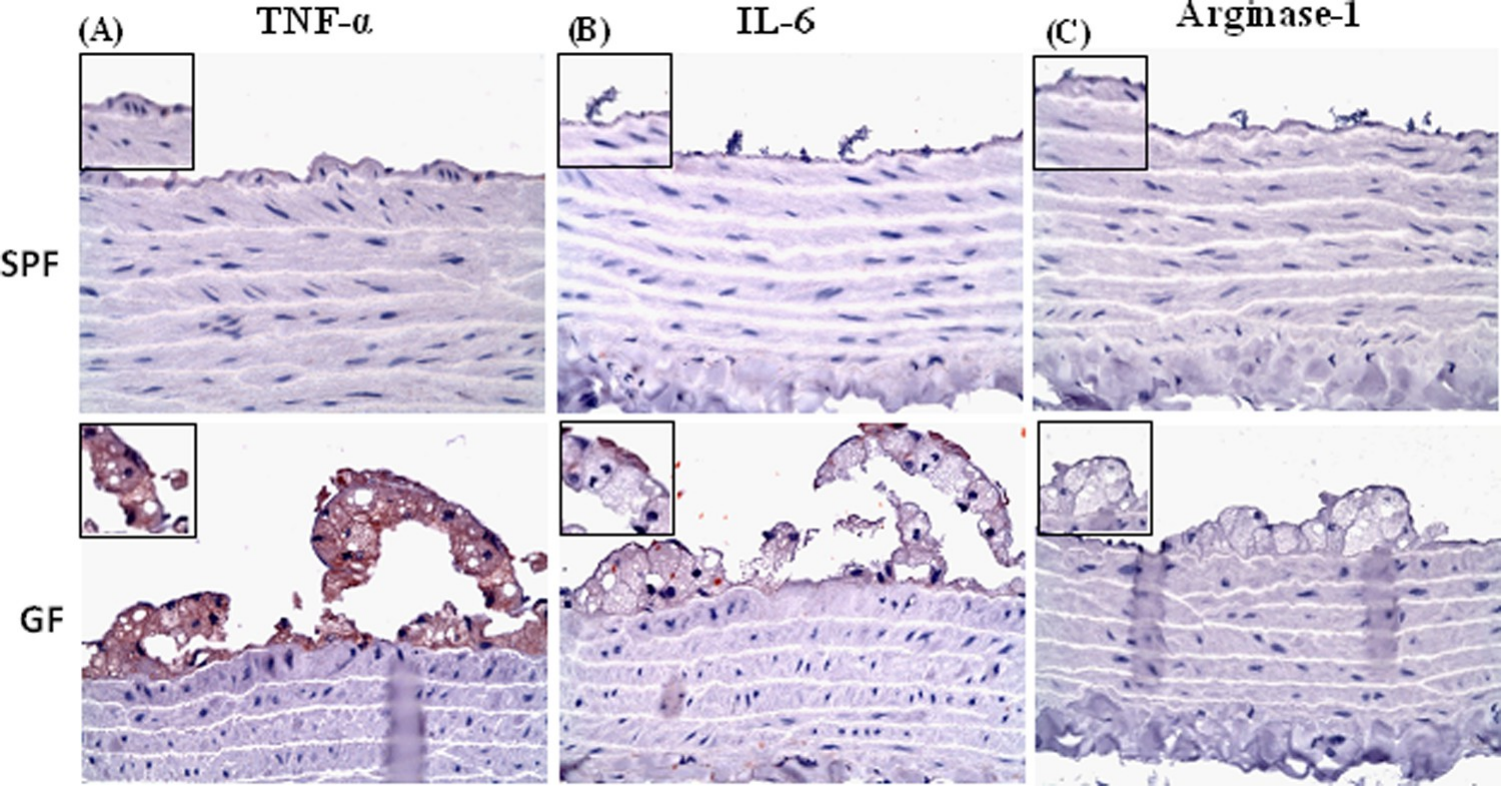
Immunohistochemical staining of thoracic aorta lesions. (A) TNF-α stain. (B) IL-6 stain. (C) Arginase-1 stain. GF: germ free, SPF: specific pathogen free.

The aortic endothelium plaque lesions in GF-ApoE^-/-^ rats initiated with endothelium injury were detected by von Willebrand factor (VWF) immunostaining at 13 weeks. VWF-positive endothelial cells were observed in atherosclerosis lesions of GF-ApoE^-/-^ rats from 13 to 52 weeks. In contrast, the SPF-ApoE^-/-^ rats showed VWF and CD31 immunostaining positive fatty streak only at 52 weeks (Fig 2B and S1 Fig). On the other hand, enrichment of CD68 positive macrophages (foam cells) was observed in the tunica intima of GF-ApoE^-/-^ rats from to 26–52 weeks, but not at 13 weeks. Several CD68 positive foam cells were observed in SPF-ApoE^-/-^ rats at 52 weeks (Fig 2C). In GF-ApoE^-/-^ rats, atherosclerotic lesions were seen not only in the vascular endothelium, but also in the tunica media (which mostly contains smooth muscle cells and elastic lamella). These morphological changes were highlighted by EMT staining. A slightly irregular arrangement of fracture elastic fibers was initially observed in the middle layer of the blood vessel wall in 26-week-old GF-ApoE^-/-^ rats, with a larger irregular arrangement detected at 52 weeks. Interestingly, lesions involving tunica media were not observed in SPF ApoE^-/-^ rats (Fig 4A). Staining with α-smooth muscle actin was used to investigate whether smooth muscle cells (SMCs) were being replaced by fibrous tissue (Fig 4B). Smooth muscle cells were replaced by amorphous pale tissue in GF-ApoE^-/-^ rats at 52 weeks, with a portion of smooth muscle actin failing to stain. Furthermore, the expression of TNF-α and IL-6 in atherosclerotic lesions at 52 weeks was detected in GF-ApoE^-/-^ rats, but not in SPF-ApoE^-/-^ rats. The expression of TNF-α and IL-6 in foam cells (where there was a predominantly positive signal found in the cytoplasm) was detected in the tunica intima. Finally, no arginase-1 signal was detected from either group at any time point (Fig 5).

### Untargeted metabolomic profiling of GF and SPF ApoE^-/-^ rats

**Fig 6:**
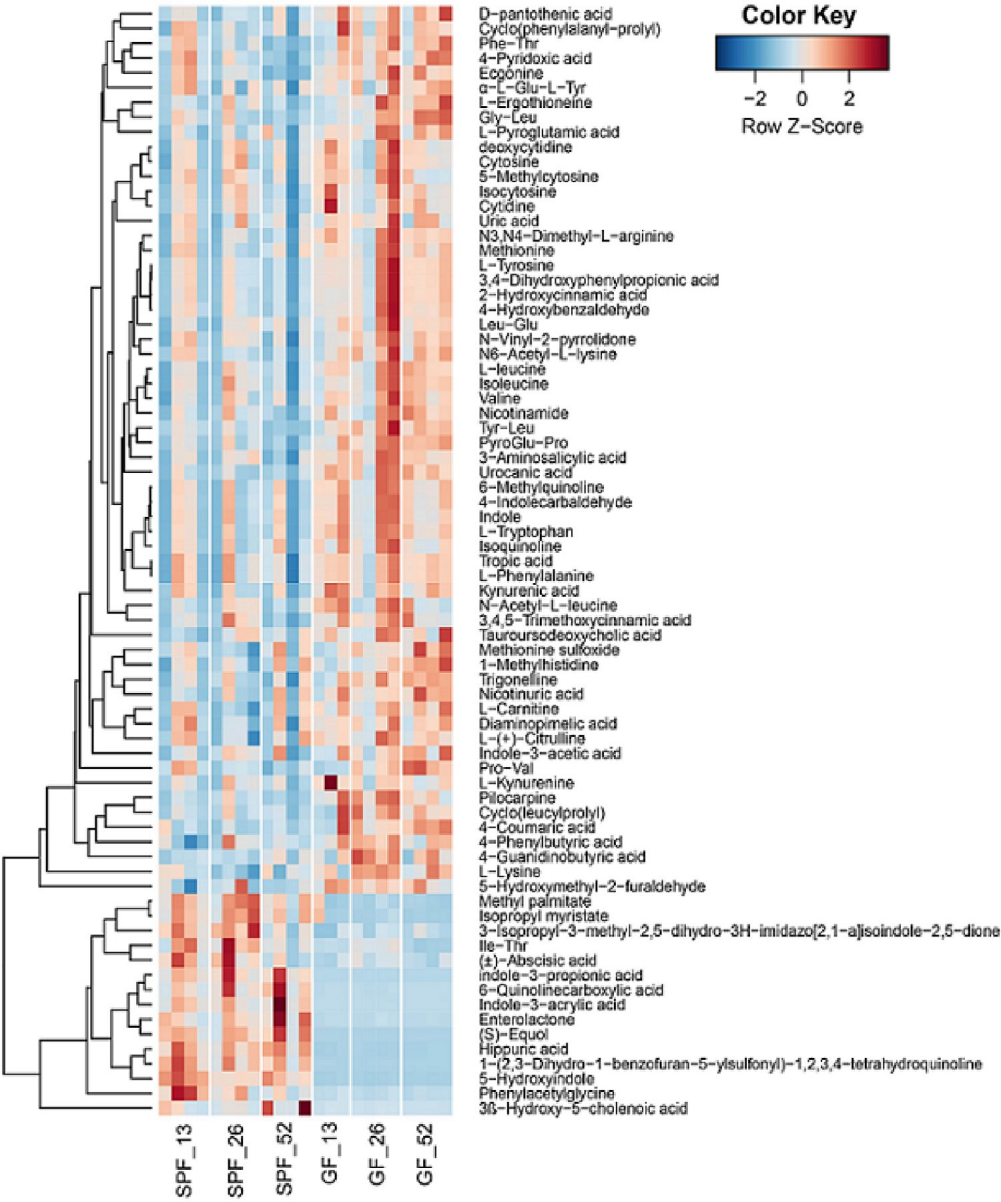
Heatmap showing the distribution of 75 metabolites that were significantly different between GF and SPF serum samples from ApoE^-/-^ rats. The list includes mass data (m/z) that could be annotated with databases, including GNPS and CD2.1. Both groups contain 13, 26, and 52 weeks old ApoE^-/-^ rats, with a total of 12 rats in the SPF group and 11 in GF group (N = 3~4/group). GF: germ free, SPF: specific pathogen free.

**Fig 7:**
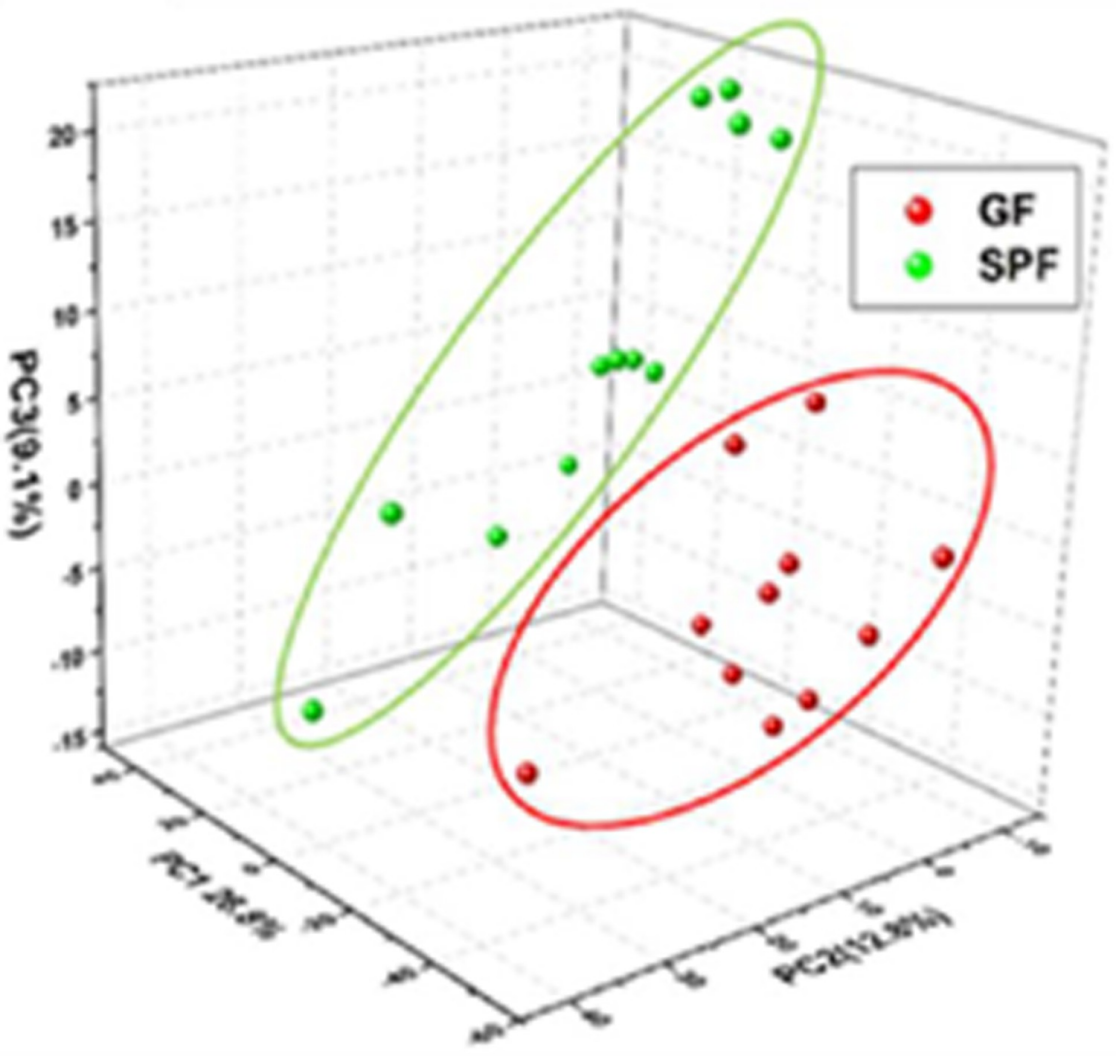
Three-dimensional principle component analysis (PCA) scores plot of serum samples in the ApoE^-/-^ rats. There are obvious differences between germ-free (GF) serum samples and specific-pathogen-free (SPF) serum samples with PC1(26.8%), PC2(12.8%), and PC3(9.1%). Regardless of age, GF rats (n = 11) and SPF rats (n = 12) were separated from each other, indicating that the metabolome profiles were different.

Gut bacteria produce small metabolites in the circulation system of their hosts; these metabolites can reach several organs of the host organism. To investigate the protective effect of gut microbiota, we performed untargeted metabolomics on serum from GF- and SPF-ApoE^-/-^ rats at 13, 26, and 52 weeks (n = 3–4/group). The LC-MS data were processed using the Compound Discoverer 2.1 and GNPS online workflow, and the two results were combined. A total of 1602 features were detected in all 23 rat serum samples, and 297 compounds were identified by searching mzCloud and (with a score threshold of 50 and 0.7). After manual validation of the MS/MS spectra, there were 75 compounds with significant (p < 0.05) differences between SPF- and GF-ApoE^-/-^ rats. These compounds were quantified and visualized as heatmaps. The heatmap exhibited different distribution patterns of metabolites between the GF- and SPF-ApoE^-/-^ groups (Fig 6 and S1 Data). PCA analyses also indicated that the serum metabolite profiles were quite diverse between SPF- and GF-ApoE^-/-^ rats (Fig 7).

### Gut microbiota-related compounds were increased in SPF-ApoE^-/-^ rats

We then looked at the significantly different (p-value < 0.05 and fold change > 2 or < 0.5) compounds between SPF- and GF-ApoE^-/-^ rats of the same age. Some compounds were increased in the SPF group, whereas others were decreased (Tables 2–4). We found nine microbial metabolites, including (S)-equol-glucuronide, indole-3-propionic acid, indole-3-acrylic acid, isoquinoline, cholic acid, 6-quinolinecarboxylic acid, hippuric acid, 5-hydroxyindole, and enterolactone-glucuronide, at significantly higher levels at different ages in SPF-ApoE^-/-^ rats than in GF-ApoE^-/-^ rats.

**Table 2:** Significantly transformed compounds in serum from 13 week-old ApoE^-/-^ rats.

Metabolites Name	Fold Change (SPF/GF)	p-value
(S)-Equol-glucuronide	440.8	8.9E-05
Indole-3-propionic acid	183.0	9.0E-05
Isoquinoline	98.3	1.1E-03
Cholic acid	70.0	2.1E-08
Indole-3-acrylic acid	53.1	9.2E-04
Asp-Leu	46.8	4.8E-03
6-Quinolinecarboxylic acid	26.4	4.7E-04
Hippuric acid	26.1	3.6E-04
5-Hydroxyindole	20.4	1.9E-09
Enterolactone-glucuronide	10.7	2.0E-02
Phenylacetylglycine	8.2	8.5E-03
Cytidine 5’-monophosphate (hydrate)	0.1	3.6E-03
Guanosine	0.1	1.3E-02

**Table 3:** Significantly transformed compounds in serum from 26 week-old ApoE^-/-^ rats.

Metabolites Name	Fold Change(SPF/GF)	p-value
(S)-Equol-glucuronide	403.1	4.9E-05
2’,2’-Difluorodeoxyuridine	555.0	3.0E-05
Indole-3-propionic acid	163.5	2.2E-05
Isoquinoline	116.1	1.2E-04
Indole-3-acrylic acid	69.8	9.1E-05
6-Quinolinecarboxylic acid	38.7	5.1E-05
Cholic acid	19.5	4.1E-06
Hippuric acid	17.2	7.0E-04
17α-Hydroxyprogesterone	13.3	1.3E-02
Corticosterone	12.5	3.3E-03
Tetrahydrocortisone	12.5	3.3E-03
5-Hydroxyindole	10.3	1.0E-07
Cortisone	9.7	2.2E-02
Enterolactone-glucuronide	9.2	1.9E-02
Cortisol	8.4	4.5E-02
L-Lysine	0.6	3.7E-03

**Table 4:** Significantly transformed compounds in serum from 52 week-old ApoE^-/-^ rats.

Metabolites Name	Fold Change(SPF/GF)	p-value
**(S)-Equol-glucuronide**	771.9	9.0E-06
**5-Hydroxyindole**	14.3	7.8E-09
**Isoquinoline**	198.7	2.9E-05
**Indole-3-propionic acid**	129.8	3.9E-05
**Indole-3-acrylic acid**	108.7	2.1E-05
**Isopropyl myristate**	79.7	3.2E-03
**6-Quinolinecarboxylic acid**	49.3	5.2E-05
**Glycochenodeoxycholic acid**	42.7	2.4E-03
**Cholic acid**	39.1	9.6E-08
**Enterolactone-glucuronide**	26.9	3.7E-04
**Hippuric acid**	22.9	3.0E-04
**PyroGlu-Pro**	0.4	7.6E-03
**Gly-Leu**	0.2	3.1E-02
**Spermidine**	0.2	1.9E-02
**5-Hydroxyindole-3-acetic acid**	0.1	9.5E-04
**Asp-Leu**	0.1	6.9E-03
**Phe-Thr**	0.1	8.1E-05
**Tyr-Leu**	0.1	1.5E-03

## Discussion

In the present study, we provide experimental evidence for the effects of gut microbiota on the development of endothelium plaque lesions in ApoE^-/-^ rats. Furthermore, a MS-based metabolomics approach was used to study the metabolic phenotype differences between SPF and GF groups. A previous study suggested that approximately 30% of metabolites detected in the human body originate from the microbiota [21]. In our study, reduction in endothelium plaque lesions in SPF- ApoE^-/-^ rats compared to that in GF-ApoE^-/-^ rats was confirmed at different time intervals, including 13, 26, and 52 weeks. Aortic atherosclerosis was mild in the SPF-ApoE^-/-^ group compared to that in the GF-ApoE^-/-^ group, and severe coronary atherosclerosis was observed in the GF-ApoE^-/-^ group at 52 weeks. A similar result was observed by Stepankova et al., where they reported the influence of gut microbiota on the development of atherosclerotic lesions in ApoE^-/-^ mice [8]. However, Kiouptsi et al. reported that GF low-density lipoprotein receptor-deficient (*Ldlr^-/-^*) mice did not reveal a significant contribution of the microbiota in late aortic atherosclerosis [22]. This is inconsistent with our results, possibly due to the different use of animal models.

We examined ApoE^-/-^ rats under GF and SPF conditions at serial experimental time points of 13, 26, and 52 weeks. In comparison to GF-ApoE^-/-^ rats, SPF-ApoE^-/-^ rats displayed limited lesion formation of atherosclerotic plaques in the thoracic aorta at 52 weeks. In contrast, exacerbated atherosclerotic lesions were observed in the coronary arteries of the GF-ApoE^-/-^ rats. Stepankova et al. reported that the absence of microbiota accelerates atherosclerosis in ApoE^-/-^ mice fed a standard low-cholesterol diet (0–0.02% w/w), however, atherogenic differences between GF and conventionally raised (Conv-R) mice were not apparent when fed a high cholesterol diet [6, 8]. Interestingly, Kasahara et al. reported that the GF ApoE^-/-^ mice caused a significant reduction in atherosclerotic lesion formation compared with Conv-R ApoE^-/-^ mice which might be associated with the attenuation of lipopolysaccharide-mediated inflammatory responses [23]. According to these researchs, we speculated that the effect of microbiota on the development of atherosclerosis might dietary dependent, especially cholesterol. Although our research, the ApoE^-/-^ rat fed low cholesterol diet (0.0275% w/w), serum cholesterol levels were similar in the GF and SPF groups. This result was different from other studies [6, 8, 23], which might be related to the age of the animals. In our study, cholesterol of serum was increased in the GF ApoE^-/-^ rats than SPF ApoE^-/-^ rats at 13 weeks. However, as animals age, this phenomenon becomes less obvious. To the best of our knowledge, this is the first report on the development of severe endothelium plaque lesions in GF-ApoE^-/-^ rats fed a standard chow diet. These findings are similar to previous observations of excess atherosclerotic plaques developed in GF-ApoE^-/-^ mice [8, 24].

In this study, we also confirmed that the earliest visible plaque lesions appeared as fibrous- and cotton-like substances in GF-ApoE^-/-^ rats at 13 weeks of age, but were not observed in the SPF rats. Fatty streaks occurred due to the accumulation of lipid-laden foam cells in the intimal layer of the thoracic aorta from to 26–52 weeks. However, only mild vascular lesions were found in SPF-ApoE^-/-^ rats. Furthermore, the GF-ApoE^-/-^ rats developed lesions that were distinct from those seen in rabbit atheroma induced by a high-cholesterol diet. The lesion area is mainly composed of smooth muscle cells and macrophages [25]. The histological results indicate that the morphological structure of atherosclerotic vulnerable plaques is more severe in GF rats. Previous reports identified three macrophage populations, termed resident-like macrophages, inflammatory macrophages, and triggering receptor expressed on myeloid cells 2 (Trem2) ^hi^ macrophages [26]. In the present study, the plaque macrophages express proinflammatory cytokines that have been assigned a proatherogenic role, such as TNF-α, IL-6, and CD68 [27]. Multiple studies have indicated an association between gut microbiota and atherosclerosis, and there is mounting evidence that the microbial metabolism of dietary nutrients influences pathophysiology. There is evidence that the gut microbiota contributes to systemic inflammation, metabolic syndrome, vascular dysfunction, and atherosclerosis. Thus, modulating gut microbes and their metabolites may have potential health benefits.

In our study, nine microbial metabolites were significantly higher in the SPF groups than in the GF groups across all ages. Indole-3-propionate (IPA) and indole-3-acrylic acid (I3A) are tryptophan metabolites secreted by gut microbiota [28]. *Clostridium sporogenes* convert tryptophan to IPA [29]. Cason et al. showed that the concentrations of plasma metabolites, including indole and indole-3-propionic acid, were negatively associated with advanced atherosclerosis [11], whereas Gesper et al. reported that the gut-derived metabolite indole-3-propionic acid was identified as a mitochondrial modulator in cardiomyocytes, and altered cardiac function in an *ex vivo* mouse model [30]. (S)-equol-glucuronide is a metabolite of (S)-equol produced by intestinal β-glucosidases from gut microbiota, including *Lactobacillus* and *Bifidobacterium* [31]. Sekikawa et al. reported that S-equol is anti-atherogenic, improves arterial stiffness, and may prevent coronary heart disease [32]. Enterolactone is a biphenol that can function as an antioxidant and has a protective effect against cardiovascular diseases. Enterolactone-glucuronide is an enterolactone metabolite produced by the gut microbiota and previous research has found that elevated serum levels of enterolactone are associated with lower mortality from CVD [33], and metabolism of cholesterol and bile acids by gut microbiota [34]. Mayerhofer et al. reported that decreased serum levels of primary bile acids and increased ratios of secondary bile acids to primary bile acids are associated with CVD [35]. Isoquinolone is an alkaloid that has multiple therapeutic effects, including hypocholesterolemic capacity and anti-inflammatory, anti-oxidative, and anti-atherosclerotic properties [36]. Studies in animal models of hyperlipidemia have supported the beneficial effects of natural alkaloids in delaying atherosclerotic progression [37, 38]. Zhang et al. published a meta-analysis that found ioquinoline alkaloids and indole alkaloids to be effective in preventing aortic atherosclerosis in ApoE^-/-^ mice [37]. Based on the evidence presented above, it is possible to conclude that gut microbes and their metabolites influence endothelial plaque lesion formation in ApoE^-/-^ rats.

Based on these findings and discussion, gut microbiota-derived metabolites have been identified as a key factor in cardiovascular health and disease. Therefore, a better understanding of the gut microbial pathways involved in the biosynthesis of plaque-related metabolites will benefit heart health management, particularly in the prevention of CVD.

## Supporting information

S1 Table Serum biochemistry of WT rats at different ages in GF and SPF rats. Values are expressed as mean ± SD. WT: wild type; T-CHO: total cholesterol; TG: triglycerides.
(DOCX)

S1 Fig The CD31 immunohistochemical staining of thoracic aorta lesions. GF: germ free, SPF: specific pathogen free.
(TIF)

S1 Data The raw data of metabolites between the GF- and SPF-ApoE^-/-^ groups. (XLSX)
