# Supplementary material for: Circulating gut microbiota-related metabolites influence endothelium plaque lesion formation in ApoE knockout rats
Source: PLoS One. 2022 May 6;17(5):e0264934. doi: 10.1371/journal.pone.0264934 (PMC9075652; doi:10.1371/journal.pone.0264934)
Supplement: S1 Table — Values are expressed as mean ± SD. WT: wild type; T-CHO: total cholesterol; TG: triglycerides. (DOCX) [file pone.0264934.s001.docx]

| **weeks** | **groups** | **T-CHO (mg/dL)** | **TG (mg/dL)** |
| --- | --- | --- | --- |
| **13** | **GF** | 83.5±18.8 | 242.5±46.6 |
|  | **SPF** | 90.5±15.8 | 298.1±57.2 |
| **26** | **GF** | 92.2±11.9 | 325.0±62.9 |
|  | **SPF** | 88.7±13.1 | 356.4±47.0 |
| **52** | **GF** | 129.8±25.0 | 328.5±59.6 |
|  | **SPF** | 117.9±27.5 | 341.5±32.8 |
