# Supplementary figures and images for: Circulating gut microbiota-related metabolites influence endothelium plaque lesion formation in ApoE knockout rats
Source: PLoS One. 2022 May 6;17(5):e0264934. doi: 10.1371/journal.pone.0264934 (PMC9075652; doi:10.1371/journal.pone.0264934)

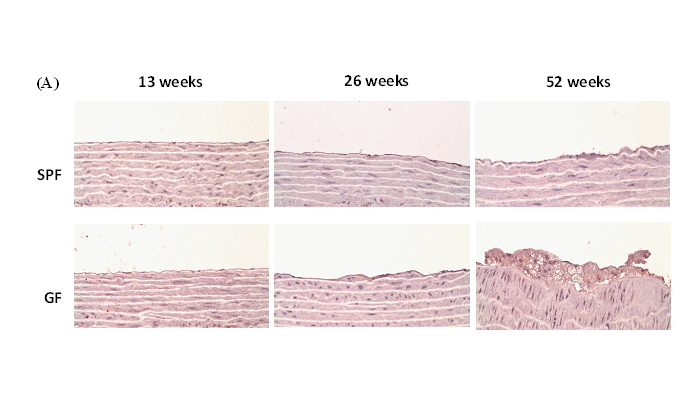

Supplement: S1 Fig — GF: germ free, SPF: specific pathogen free. (TIF) [file pone.0264934.s002.tif]
